# Rural nurses’ antiretroviral prescribing practices in children, Limpopo province, South Africa

**DOI:** 10.4102/sajhivmed.v24i1.1470

**Published:** 2023-07-07

**Authors:** Linneth N. Mabila, Patrick H. Demana, Tebogo M. Mothiba

**Affiliations:** 1 Department of Pharmacy, Faculty of Health Sciences, University of Limpopo, Polokwane, South Africa; 2 School of Pharmacy, Faculty of Health Sciences, Sefako Makgatho Health Sciences University, Pretoria, South Africa; 3 Faculty of Health Sciences, University of Limpopo, Polokwane, South Africa

**Keywords:** antiretroviral therapy, ART, children, nurse prescriber, prescribing practices, prescribing errors, antiretroviral stewardship

## Abstract

**Background:**

Errors in antiretroviral therapy (ART) use are common in children living with HIV (CLHIV), but there is limited evidence from rural primary healthcare (PHC) facilities where trained professional nurses initiate and manage most CLHIV.

**Objectives:**

To assess antiretroviral prescribing practices of trained professional nurses in Mopani District’s rural facilities and compare them to the 2015 national consolidated guidelines to evaluate the appropriateness of ART use.

**Method:**

A four-year (2015–2018) retrospective cross-sectional medical record review was conducted of CLHIV in 94 rural PHC facilities of Mopani District. Inclusion criteria were: age under 15 years, initiated on ART by nurses in 2015 and virally unsuppressed (viral load ≥ 400 copies/mL) by the end of December 2018.

**Results:**

A total of 16 669 antiretrovirals were prescribed from 7035 clinic visits. A correct ART regimen and dosage form was prescribed in 7045 (96%) and 15 502 (93%) of the cases. However, errors were common: 2928 (23%) incorrect doses, 15 502 (93%) incorrect dosing frequencies, and 4122 (61%) incorrectly dispensed antiretrovirals, and 3636 (28%) incorrect dosing frequencies.

**Conclusion:**

Antiretroviral prescribing errors in the form of drug omissions in ART regimens, incorrect dosing and dosing frequencies, lack of formulation considerations, and inadequate monthly supplies of antiretrovirals were commonly observed in this review. Antiretroviral stewardship programmes should be considered to develop and establish a fundamental strategy for improving quality in managing CLHIV.

**What this study adds:** A high prevalence of prescribing and dispensing errors in managing children on ART by trained nurses in rural PHC facilities was observed.

## Introduction

In South Africa (SA), since 2010, nurses have taken on various HIV care tasks, such as HIV diagnosis and prescribing antiretroviral treatment (ART), adopting the task-shifting approach developed by the World Health Organization (WHO).^1,2^ This decision led to the decentralisation of ART services to rural primary healthcare (PHC) facilities, where professional nurses were trained and capacitated in the Nurse-Initiated Management of ART (NIMART) programme.^3,4,5,6,7,8,9^ Challenges have been identified with NIMART training and implementation in PHC facilities.^6^ There are many other challenges in achieving and maintaining virological suppression in children on ART. Despite these challenges, the scale-up of ART programmes has considerably improved survival in children living with HIV (CLHIV) in SA.^10,11^

Antiretroviral treatment failure in children has received insufficient attention in managing CLHIV,^12,13^ which results in the development of resistance to antiretrovirals.^13,14^ Antiretroviral therapy failure rates among children in poor, resource-limited settings have recently been shown to have escalated from 19.3% to over 32%.^15,16^

Excellent adherence to ART is required in order to achieve and maintain virological suppression, but this is challenging in CLHIV, especially in adolescents.^17,18,19,20,21,22,23,24^

Prescribing and dispensing errors are the most significant cause of medication errors. They occur in general clinical practice as well as in hospitals and clinics.^25,26,27,28^ They can affect patient safety and healthcare quality.^26^

To our knowledge, there is currently limited published research and evaluations of antiretroviral prescribing practices in children under 15 years initiated and managed on ART by NIMART-trained professional nurses in resource-limited rural PHC facilities. This study aimed to evaluate the appropriateness of antiretroviral use and describe the compliance of NIMART-trained nurses with HIV and/or AIDS treatment guidelines.

## Research methods and design

This was a four-year (01 January 2015 to 31 December 2018) descriptive, cross-sectional medical record study to determine NIMART-trained professional nurses’ ART prescribing practices in rural PHC facilities of the Mopani District in Limpopo province, South Africa.

### Study setting

The study was conducted in 94 rural PHC facilities, comprised of eight community health centres and 86 clinics in the Mopani District. These facilities were selected because they were accredited to roll out antiretrovirals in 2015 and had children on ART in their programme. Mopani municipality comprises 354 rural villages and 16 urban areas. Of the total district population, 81% live in rural villages, 14% in urban areas and 5% in agricultural areas. The district is divided into five local municipalities: Ba-Phalaborwa, Greater Giyani, Greater Letaba, Greater Tzaneen and Maruleng.^29^

### Study population and sampling strategy

The study population comprised children under the age of 15, initiated and managed on ART at PHC facilities of Mopani District in 2015 and virally unsuppressed (viral load [VL] ≥ 400 copies/mL) by 31 December 2018. A census data collection technique (total population purposive sampling) was used to obtain a complete picture of the problem in these PHC facilities.^30^ First, the district TIER.Net system was used to generate a list of all children under 15 years initiated on ART in 2015 (*n* = 516) and those virally unsuppressed by 31 December 2018 (*n* = 255). Second, a physical census of all 255 medical records was conducted in the facilities.

### Data collection

The principal investigator physically went to all the PHC facilities for data collection. A data collection checklist designed for this study was used to extract demographic and baseline clinical data from the medical records including age at ART initiation, gender, WHO clinical staging at ART initiation, concomitant diseases at ART initiation, and the ART regimen at initiation. The data collection checklist also captured the longitudinal ART treatment history, the prescribed dose (strength) for each drug in the regimen, the prescribed dosage form for each drug in the regimen, the prescribed dosing frequency for each drug in the regimen, and the quantity or amount dispensed for each prescribed drug in the regimen.

### Data analysis

The data collected were entered into a Microsoft Excel^TM^ spreadsheet and cleaned and imported into the Statistical Package for Social Sciences (SPSS). The mean, median, mode, and standard deviation descriptively summarise the patient’s demographic and clinical characteristics. Categorical variables, such as gender, were selected through frequency counts and percentage calculations. For continuous variables, such as age, minimum and maximum values were determined.

Similar to studies conducted by Grossberg et al.^31^ and Fairley et al.,^32^ adherence to ART was assessed by the timeliness of clinic attendance. Actual dates for clinic visits were compared with scheduled appointments for every patient. To this end, the researcher cumulatively determined the days each patient was late for ART treatment over the four years. This variable was standardised by determining its quotient from the months each patient received ART. Guided by the consolidated HIV and/or AIDS treatment guidelines, prescriptions were then analysed, evaluating the appropriateness of the prescribed ART regimen, the dose, dosage form, dosing frequency, and the quantity of monthly treatment supplied.

### Ethical considerations

Ethical approval was obtained from the University of Limpopo’s Turfloop Research Ethics Committee (TREC), certificate number TREC/81/2019: PG. Permission to conduct the study was granted by the Limpopo Department of Health, approval number LP_20190, and the Department of Health Mopani District Municipality (reference: S4/2/2).

## Results

From the census of the 255 medical records of the children under study, 7351 analysable visits were obtained. The baseline characteristics of the 255 CLHIV are shown in Table 1 at ART initiation. The majority were of school-going age. Most children (58%) were initiated on ART while in WHO Clinical Stages 1 and 2, with 28% (*n* = 72) of children without WHO Clinical Stage information. Of the 255 children, only 40 had notable concomitant diseases at initiation, such as pulmonary tuberculosis, fungal skin infections, and gastrointestinal problems.

**TABLE 1:** Baseline characteristics of virally unsuppressed children initiated on antiretroviral treatment in 2015.

Category	Frequency (*n*)	Percentage (%)
**Gender distribution at ART initiation (*n* = 255)**		
Female	132	51.76
Male	123	48.24
**Age distribution at ART initiation (*n* = 255)**		
Newborns (0–2 months)	26	10.20
Infants (3–12 months)	14	5.49
Toddlers (13–23 months)	30	11.76
Pre-scholars (2–5 years)	32	12.55
School-aged (6–14 years)	153	60.00
**WHO Clinical Stage at ART initiation (*n* = 255)**		
Stage 1	101	39.61
Stage 2	28	10.98
Stage 3	6	2.35
Stage 4	6	2.35
Unknown	72	28.24
**Notable concomitant diseases at ART initiation (*n* = 255)**		
Yes	40	16.00
No	195	76.00
Unknown	20	8.00
**Nature of concomitant diseases (*n* = 40)**		
Pulmonary tuberculosis	8	15.69
Skin fungal infection	8	15.69
Severe acute malnutrition	6	11.76
Gastrointestinal	4	7.84

ART, antiretroviral treatment; WHO, World Health Organization.

Table 2 gives an overview of the ART regimens the children were initiated on per sub-district. Most children (78%) were initiated on an Abacavir-containing regimen, as recommended by the 2015 South African HIV/AIDS guidelines, but 7% of children were initiated on Stavudine-containing regimens.

**TABLE 2:** Antiretroviral treatment initiation by sub-district and antiretroviral treatment regimens.

Category	Frequency (*n*)	Percentage (%)
**ART initiations per sub-district municipality (*n* = 255)**		
Greater Tzaneen	88	34.51
Greater Giyani	51	21.00
Greater Letaba	47	18.43
Ba-Phalaborwa	35	13.73
Greater Maruleng	34	13.33
**ART regimens at initiation (*n* = 255)**		
ABC + 3TC + EFV	122	47.84
ABC + 3TC + LPV/r	82	32.16
ABC + 3TC + NVP	1	0.39
d4T + 3TC + EFV	11	4.31
d4T + 3TC + LPV/r	6	2.35
TDF + FTC + EFV	8	3.14
AZT + 3TC + EFV	7	2.75
AZT + 3TC + LPV/r	4	1.57
3TC + EFV + LPV/r	1	0.39
Regimen not specified†	13	5.10
**Summary of regimens prescribed at initiation in 2015 (*n* = 7351)**		
Triple regimen	7045	95.84
Dual regimen	65	0.89
Mono regimen	15	0.20
Quad regimen	3	0.04
Unknown	223	3.03

ART, antiretroviral treatment; ABC, Abacavir; 3TC, Lamivudine; EFV, Efavirenz; LPV/r, Lopinavir/ritonavir; NVP, Nevirapine; d4T, Stavudine; TDF, Tenofovir; FTC, Emtricitabine.

† , The regimen at initiation was not specified in the medical record.

In 96% of the prescriptions, children were prescribed a triple regimen of ART by the nurses, as recommended by the South African HIV/AIDS treatment guidelines. In three cases the nurses prescribed four antiretrovirals instead of the three recommended by the treatment guidelines by prescribing Lamivudine in addition to the Abacavir/Lamivudine fixed-dose combination.

The nurses’ ART prescribing practices and medication dispensed are shown in Table 3. The majority (93%) of the antiretrovirals were prescribed in a correct dosage form suitable for the children’s weight and age. The reasons for the 1192 incorrect prescriptions included: incomplete prescription (*n* = 928), incorrect doses (*n* = 106), and illegible (*n* = 102). Dosing frequency was not indicated in 22% of prescriptions.

**TABLE 3:** Summary of Nurse-Initiated-Management of Antiretroviral Treatment-trained nurses’ antiretroviral treatment prescribing practices for children living with HIV and antiretroviral treatment supplied.

Category	Frequency (*n*)	Percentage (%)
**Prescriptions prescribed the correct dosage form (*n* = 16 694)**		
Yes	15 502	92.86
No	1192	7.14
**Prescriptions with an indication of a dosing frequency (*n* = 16 694)**		
Yes	13 054	78.20
No	3640	21.80
**Prescriptions indicating the strength prescribed (*n* = 16 694)**		
Yes	12 467	74.67
No	4227	25.33
**Compliance of prescribed strength to ART guidelines (*n* = 12 467)**		
Correct strength/dose	9539	76.51
Medication errors	2928	23.49
**Observed medication errors (*n* = 2928)**		
Overdosing	2025	69.15
Underdosing	903	30.85
**Quantity dispensed indicated (*n* = 15 502)**		
Yes	7449	48.05
No	8053	51.95
**Appropriateness of the monthly treatment supplied (*n* = 7449)**		
Adequate supply	2883	38.70
Under supply	2927	39.29
Oversupply	1195	16.04
Unknown	444	5.96

ART, antiretroviral treatment.

A common reason for incorrectly prescribed dosage forms was related to Efavirenz (EFV) 50 mg stockouts – nurses prescribed EFV 600 mg enteric-coated tablets for children in the 300 mg weight band, and caregivers were instructed to administer half of the tablet even though the guidelines advise against chewing or crushing this form of a tablet.

Medication dosage errors were more commonly an overdose than an underdose of their ART regimen (defined by Dakshina et al.^33^ as antiretroviral drugs prescribed at a dose higher or lower than the dose recommended in the 2015 South African Consolidated Treatment Guideline for the child’s weight band).

The amount of ART dispensed could be assessed in 15 502 cases (missing values due to missing data on weight, dose, dose frequency, or drug prescribed). Nurses only indicated the amount of ART dispensed in 48% of prescribed antiretrovirals (Table 3). From the 7449 antiretrovirals with an indication of the quantity dispensed, only 39% (*n* = 2883) of the children were dispensed an adequate amount of ART to last them until their next appointment date and 16% (*n* = 1195) of the antiretrovirals were over-supplied.

Table 4 gives details of the correctness of the dosing frequencies from the 12 647 prescriptions with a dosing frequency indicated.

**TABLE 4:** Correctness of prescribed dosing frequencies.

Recommended dosing frequencies	Twice daily		Once daily		Morning		At night		Grand total (%)
	*n*	%	*n*	%	*n*	%	*n*	%	
Twice daily	6768	67.08	1477	14.64	9	0.09	1835	18.19	77.00
At night	121	4.17	191	6.58	0	0.00	2591	89.25	22.00
Once daily	3	4.84	24	38.71	0	0.00	35	56.45	0.00

**Grand total**	**6892**	**52.80**	**1692**	**12.96**	**9**	**0.07**	**4461**	**34.17**	**100.00**

## Discussion

In this medicine utilisation review, the following forms of medication errors and prescribing irrationalities were documented: (1) antiretroviral omissions in 4% of the prescriptions, (2) 7% incorrectly prescribed dosage forms, (3) 23% incorrectly prescribed doses, (4) 28% incorrectly prescribed dosing frequencies, and (5) 61% of inadequately supplied monthly treatments.

These findings are similar to those in other studies. Mulema et al.^34^ in Uganda reported the prevalence of irrational prescribing was high among children under 15 years. Sterling^35^ reported that dosing errors, such as inappropriate dosages or frequency of administration like underdosing or overdosing, are the most common medication errors in the management of children. Other noted medication errors include the incorrect selection of the indication, incorrect route of administration, and failure to screen drug interactions or monitor side effects.^36,37^

Simonsen et al.^38^ highlight that nurses experience an insufficient pharmacological knowledge of medication, mainly in dosage calculations and medicine management. In this study we see the high prevalence of irrational prescriptions in this review of children as an indication that there is a need to make NIMART-trained nurses aware of the importance of rational prescribing in children on ART since their failure to prescribe ART according to treatment guidelines is an irrational use of antiretrovirals.^27,39^

Irrational prescribing often results from wrong medical decisions associated with the lack of knowledge, skill or inadequate training.^27,36,40,41,42^ Also, strenuous working conditions, complex or unclear guidelines and insufficient communication between health professionals such as nurses and doctors have been identified as contributing to prescribing errors.^27,43,44,45^ Since clinical nursing knowledge is the key to quality patient care,^46^ it is, therefore, crucial for NIMART-trained nurses to have an adequate understanding of the relevant aspects of antiretroviral (ARV) use to address issues of irrational antiretroviral use.

**FIGURE 1:**
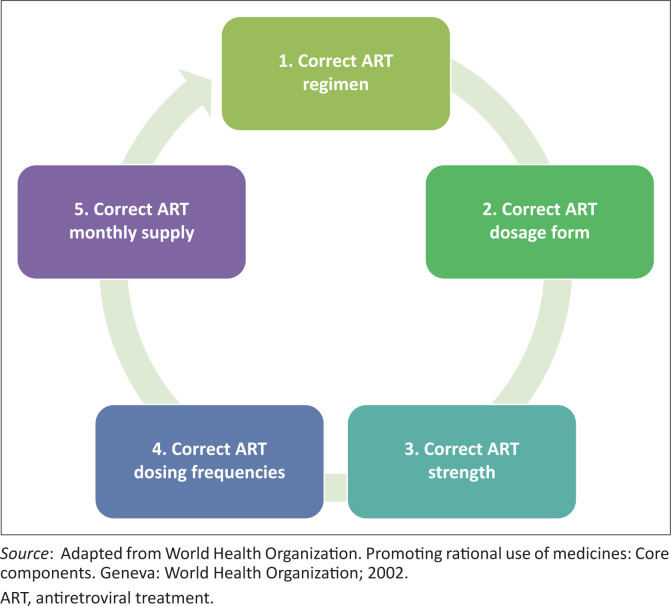
Visual illustration of the rational antiretroviral therapy prescribing cycle.

Nurse-Initiated-Management of ART-trained nurses should follow the proposed rational ART prescribing cycle and always prescribe for children initiated and managed on ART (1) a correct triple regimen as recommended in recent HIV and AIDS treatment guidelines, (2) the correct dosage form mindful of their age and drug administration properties, (3) the correct strength suitable for their body weight that avoids cases of underdosing or overdosing, and (4) correct dosing frequency that takes into consideration the pharmacokintetics (PKs) and pharmacodynamics (PDs) aspects of the antiretrovirals prescribed (see Figure 1^47^ for visual emphasis).

Our findings indicate that education, training, mentorship, and support should be targeted at all nurses who have ART prescribing responsibility for children, which aims at empowering them with the knowledge of (1) which antiretroviral tablets should be swallowed whole, (2) which ones cannot be crushed, divided, or chewed, (3) which ones are 12 hourly or 24 hourly formulations, and (4) the correct quantity of all prescribed antiretrovirals to last them until their next scheduled appointment to ensure that they do not run out of treatment as this could predispose the children to the development of drug resistance and ultimately treatment failure.

This study has limitations. First, this study was conducted in 94 of 108 rural PHC clinics of one district municipality out of five districts in the Limpopo province. Therefore, the results of this study may not be generalisable to urban areas or settings. Second, the study was cross-sectional and thus could not evaluate the relationship between incorrect antiretroviral dosing and virological outcomes or whether treatment discontinuations or adverse effects were associated with overdosing. Third, the study only reviewed the prescribing practices of virologically unsuppressed children (VL ≥ 400 copies/mL) initiated on ART and we did not review prescribing practices of virally suppressed children. Therefore, we cannot comment on whether these findings are comparable with prescribing practices in virally suppressed children.

## Conclusion

From the findings of this antiretroviral utilisation review, there is evidence of the prevalence of ART prescribing errors in the form of drug omissions in antiretroviral regimens. In addition, there were incorrect dosing and dosing frequencies, a lack of formulation considerations, and inadequate monthly supplies of antiretrovirals. The observed irrationality of the NIMART-trained nurse’s prescribing practices in this review depicts a lack of compliance with HIV and AIDs treatment guideline recommendations.

We consider the high prevalence of prescribing errors in this review as an indication that there is a need to train NIMART-trained nurses on the importance of rational prescribing in children on ART since the failure to prescribe ART according to treatment guidelines results in ART medication errors.^48^ Therefore, tackling the prescribing and medication errors in children should be prioritised to improve healthcare delivery towards ensuring patient safety and allowing for optimal utilisation of antiretrovirals. It is standard practice during nursing education to receive instructions on a guide to clinical medication administration and the protection of patient safety known as the ‘five rights’ or ‘five Rs’ of medication administration.^49,50^ In PHC settings, nurses have a role to play in the promotion of the rational medicine use.^51^ Hence, for successful treatment outcomes in children, professional nurses need to perform their ART dosing and dispensing roles as guided by the HIV and AIDS guidelines to ensure the rational use of antiretrovirals in managing children.

The rational use of antiretrovirals should be an adopted strategy for improving treatment outcomes.^52^ A pharmacist-led ART stewardship programme, as well as dosing and dispensing continuous professional development training programmes promoting the appropriate use of antiretrovirals in children on ART in resource-limited rural PHC clinics, should be a considered intervention to give special attention to the rational use of antiretrovirals in children especially now that antiretroviral resistance is currently becoming a significant rural health problem in CLHIV.
